# Emerging Anthelmintic Resistance in Poultry: Can Ethnopharmacological Approaches Offer a Solution?

**DOI:** 10.3389/fphar.2021.774896

**Published:** 2022-02-14

**Authors:** Gerald Zirintunda, Savino Biryomumaisho, Keneth Iceland Kasozi, Gaber El-Saber Batiha, John Kateregga, Patrick Vudriko, Sarah Nalule, Deogracious Olila, Mariam Kajoba, Kevin Matama, Mercy Rukundo Kwizera, Mohammed M. Ghoneim, Mahmoud Abdelhamid, Sameh S. Zaghlool, Sultan Alshehri, Mohamed A. Abdelgawad, James Acai-Okwee

**Affiliations:** ^1^ School of Veterinary Medicine and Animal Resources, Makerere University, Kampala, Uganda; ^2^ Infection Medicine, Deanery of Biomedical Sciences, College of Medicine and Veterinary Medicine, University of Edinburgh, Scotland, United Kingdom; ^3^ School of Medicine, Kabale University, Kabale, Uganda; ^4^ Department of Pharmacology and Therapeutics, Faculty of Veterinary Medicine, Damanhour University, Albeheira, Egypt; ^5^ Department of Animal Production and Management, Faculty of Agriculture and Animal Sciences, Busitema University, Soroti, Uganda; ^6^ School of Pharmacy, Kampala International University Western Campus, Bushenyi, Uganda; ^7^ Biology Department, Faculty of Applied Sciences, Umm Al-Qura University, Makkah, Saudi Arabia; ^8^ Department of Parasitology, Faculty of Veterinary Medicine, Aswan University, Aswan, Egypt; ^9^ Pharmacology and Toxicology Department, Faculty of Pharmacy, Modern University for Technology and Information, Cairo, Egypt; ^10^ Department of Pharmaceutics, College of Pharmacy, King Saud University, Riyadh, Saudi Arabia; ^11^ Department of Pharmaceutical Chemistry, College of Pharmacy, Jouf University, Al Jouf, Saudi Arabia

**Keywords:** synthetic, toxicity, safety, medicine, ethnoveterinary, parasites, nematodes, plant

## Abstract

Limited pharmacological studies have been conducted on plant species used against poultry helminths. The objective of this study was to provide a basis for plant based anthelmintics as possible alternatives against poultry anthelmintic resistance. The study justified the need for alternative anthelmintics. The study places emphasis on the increasing anthelmintic resistance, mechanism of resistance, and preparational protocols for plant anthelmintics and their associated mechanism of action. Pharmaceutical studies on plants as alternative therapies for the control of helminth parasites have not been fully explored especially in several developing countries. Plants from a broad range of species produce a wide variety of compounds that are potential anthelmintics candidates. Important phenolic acids have been found in *Brassica rapa L*. and *Terminalia avicenniodes Guill. and Perri* that affect the cell signaling pathways and gene expression. Benzo (c) phenanthridine and isoquinoline alkaloids are neurotoxic to helminths. Steroidal saponins (polyphyllin D and dioscin) interact with helminthic mitochondrial activity, alter cell membrane permeability, vacuolation and membrane damage. Benzyl isothiocyanate glucosinolates interfere with DNA replication and protein expression, while isoflavones from *Acacia oxyphylla* cause helminth flaccid paralysis, inhibit energy generation, and affect calcium utilization. Condensed tannins have been shown to cause the death of nematodes and paralysis leading to expulsion from the gastro-intestinal tract. Flavonoids from *Chenopodium album L* and *Mangifera indica L* act through the action of phosphodiesterase and Ca^2+^-ATPase, and flavonoids and tannins have been shown to act synergistically and are complementary to praziquantel. Artemisinins from *Artemisia cina O. Berg* are known to disrupt mitochondrial ATP production. Terpenoids from *Cucurbita moschata L* disrupt neurotransmission leading to paralysis as well as disruption of egg hatching. Yeast particle encapsulated terpenes are effective for the control of albendazole-resistant helminths.

## 1 Introduction

Ethnoveterinary medicine is an established practice, however, information on the pharmacology of plant anthelmintics for use in poultry is scarce. In Africa, the absence of pharmacovigilance policies and a lack of research on the pharmacology of plants for use in the control of helminths continues to impede innovation in this field.

Domesticated birds including turkeys, chickens, geese, and ratites are generally referred to as poultry in the United States while in Europe, poultry also includes domesticated birds kept for the benefit of humans in production (Patel et al., 2018). Productivity in poultry is compromised by an emerging helminthic burden with birds being affected by a variety of nematodes, cestodes, and trematodes (Table 1) (Ola-Fadunsin et al., 2019). Many drugs are available for the helminth control including benzimidazoles, macrocyclic lactones, and imidazothiazoles (Patel et al., 2018). Most anthelmintics exert their effects by either stunting or killing helminths. The large diversity of helminths causing parasitic infections in poultry is a challenge. There is a growing interest in the application of plant-based anthelmintics in poultry, as they are considered safer than synthetic compounds. Plant alternatives offer a cheap natural resource and some plant-based anthelmintics are more effective than synthetic anthelmintics (Karumari et al., 2014). Plant-based medicinal compounds however have unknown safety profiles and plant phytochemical composition is highly variable. There is a need to identify the bioactive compounds in ethnoveterinary products used against helminths to guide pharmacognosy and policy development.

**TABLE 1:** Common poultry helminths and major predilection sites.

Type	Species	Predilection
Nematodes	*Strongyloides avium*	Caecum
	*Trichostrongylus tenuis*	Small intestine and caecum
	*Syngamus trachea*	Trachea
	*Hetarakis gallinarum*	Caecum
	*Heterakis isolonche*	Caecum
	*Heterakis dispar*	Caecum
	*Ascaridia galli*	Small intestine
	*Eucoleus annulatus*	Mucosa of Crop and oesophagus
	*Eucoleus contorta*	Crop and oesophagus
	*Capillaria obsignata*	Small intestines
	*Capillaria anatis*	Caecum and Small intestines
	*Capillaria caudinflata*	Small intestines
	*Cheilospirura hamulosa*	Gizzard
	*Gonngylonema ingluvicola*	Crop, Oesophagus and Proventriculus
	*Tetrameres americana*	Glands of Proventriculus
	*Allodapa suctoria*	Caecum
Cestodes	*Raillietina cesticillus*	Small intestines
	*Raillientina echinobothrida*	Small intestines
	*Raillientina tetragona*	Posterior half of Small Intestines
	*Choanotaenia infundibulum*	Anterior Small intestines
	*Hymenolepis carioca*	Small intestines
	*Hymenolepis cantaniana*	Small intestines
	*Amoebotaenia cuneata*	Small intestines
	*Metroliasthes lucida*	Small intestines
	*Davainea proglottina*	Duodenum
	*Cotugnia digonopora*	Small intestines
Trematodes	*Zygocotyle lunata*	Caecum
	*Postharmostomum commutatum*	Caecum
	*Notocotylus imbricatus*	Caecum
	*Prosthogonimus anatinus*	Oviduct
	*Echinostoma cinetorchis*	Liver and intestines
	*Hypoderaeum conoideum*	Posterior Small Intestines
	*Echinoparyphium recurvatum*	Duodenum

## 2 Methods

A scoping review approach was used to get evidence on use of plants to control helminths (Sucharew and Macaluso, 2019). Structured searches were done to get information on the use of plant medicines to treat helminths in poultry. The scope of the literature included opportunities of plants used in livestock and not documented as already used in poultry against helminths. Electronic databases of Medline, PubMed, Embase, CABI Abstracts using the ovid interface, Web of Science, Scorpus were used to access published articles while google scholar was used on grey literature. The searches were done using keywords i.e., Ethnobotany, ethnopharmacology, plant anthelmintics, poultry helminths, poultry anthelmintic resistance, poultry anthelmintics, ethnoveterinary medicine. The research questions were; what plant anthelmintics are available against poultry helminths? Can plant anthelmintics be an alternative to synthetic anthelmintics? Are plant anthelmintics an answer to synthetic anthelmintic drug resistance? Literature on use of plant anthelmintics in poultry were included. Data were extracted into tables and then discussed as narrative sections in an effort to address the research questions.

## 3 Results

### 3.1 Ethnobotany in Anthelmintic Control

Plant alternatives offer an attractive option in organic farming of poultry products, lowering the poultry production costs. Many plant species have been tested for their efficacy against helminths (Table 2). For example, ginger and curcumin extracts were fairly effective by paralyzing *Ascaridia galli* after 48 h of exposure (Bazh and El-bahy, 2013). Garlic (*Allium sativum L.*) also showed anthelmintic activity (Landman, 2005). Aqueous and ethanolic extracts of *Areca catechu* reduced parasitic infestation by *Ascaridia galli* in poultry (Mubarokah et al., 2019). The latex from papaya fruits and sap also showed anthelmintic activity in chickens against *Ascaridia galli* and *Heterakis gallinae* infections (Bilandz et al., 2018).

**TABLE 2:** Major ethnoveterinary options for control of helminths, preparation, composition and target vector species. Leaves, roots and stems have been used on limited helmintic species, however studies on their pharmacology remain scarce. Information on effective dosage, effective concentration and safety are still unknown.

Plant Family	Plant botanical name	Plant Common name	Plant part used	Method of Preparation	Composition	Tried in poultry	Poultry Helminths model or other models	References
*Aloeceae*	*Aloe buettneri A.Berger*	Burn Aloe	Leaves	Leaf juice	Tannins	No		Ibrahim, Nwude, Ogunsusi, and Aliu, (1984)
					Saponins			
					Flavonoids			
*Amaranthaceae*	*Dysphania ambrosiodes (L.) Mosyakin and Clemants*	Worm grass	Leaves, Roots	Steam distillation of juice, Methanol extract	Stigmasterol	No		(MacDonald et al., 2004; Shah & Khan, 2017)
					B-sitosterol			
					Scopoletin			
	*Chenopodium album L.*	Lambsquarters	Whole Plant	Aqueous Methanol extract of powder	Alkaloids	No		(Jabbar et al., 2007; Lans and Turner, 2011)
					Saponins			
					Phenolics			
					Flavonoids			
*Amaryllidaceae*	*Allium sativum L.*	Garlic	Cloves	Decoctions or Macerates in water	Alliin	No		(Urban et al., 2008; Martins et al., 2016; Raza et al., 2016)
					Ajoenes	Yes	*Ascaridia galli*	Velkers et al. (2011)
					Allyl sulphides	No		Calzetta et al. (2020)
					1,2 vinyldithiin			
*Anacardiaceae*	*Mangifera indica L.*	Mango	Leaves,	Alcohol and water extracts	Polyphenolics, Flavonoids, triterpenoids, tannins and gallic acid	No		(Githiori et al., 2005; Shah et al., 2010)
			Bark					
			Flowers					
			Roots					
	*Anacardium occidentale L.*	Cashew tree	leaves	Acetone solvent extraction	Sugars	No		(Chota et al., 2010; Ademola and Eloff, 2011)
					Carotenoids			
					Ascorbic acid			
*Annonaceae*	*Annona senegalensis Pers.*	African custard apple	Whole plant	Aqueous extract	Triterpenes	No		(Ibrahim et al., 1984; Mustapha, 2013)
					Anthocyanes			
					Coumarins			
					Alkaloids			
*Apiaceae*	*Centalla asiatica (L.) Urb.*	Asiatic pennywort	Stalk	Methanolic extract	Alkaloids	No	*Earthworms*	(Tandon et al., 2011; Aftab et al., 2017)
					Saponins			
					Tannins			
					Phlobatannins			
					Glycosides			
*Araceae*	*Lasia spinosa (L.) Thwaites*	Lasia	Stalk	Methanolic extract	Polyphenols	No	*Earthworms*	Tandon et al. (2011)
			Leaves		Tannins			
*Arecaceae*	*Areca catechu L.*	Betel nut	Fruit	Water extract, Alcohol extract	Alkaloids	Yes	*Ascaridia galli*	(Mubarokah et al., 2019; Ozaraga et al. (2017)
					Phenols			
					Tannins			
					Flavonoids			
					phytosterols			
*Asparagaceae*	*Agave sisalana Perrine*	Sisal hemp	Leaves	Water extract of waste from decortication machine	Phlobatannins	No		(Mwale and Masika, 2009; Ade-Ajayi et al., 2011; Botura et al., 2011; Mwale and Masika, 2015)
					Terpenoids	Yes	*Heterakis gallinarum*	Mwale and Masika, (2015)
					Tannins			
					Flavonoids			
*Asphodelaceae*	*Aloe secundiflora Engl.*	Aloe	Leaves, Stem barks	Methanol extract	Tannins, Phenols, Flavonoids, Saponins, alkaloids	Yes	*Ascaridia galli*	(Kaingu et al., 2013; Abdirahman et al., 2015; Raza et al., 2016)
	*Aloe ferox Mill.*	Alligator jaw aloe	Leaves	Aloesin, Aloeresin C		No		(Mwale and Masika, 2009; Maphosa et al., 2010)
				Aloeresin A, Aloin A, Aloin B, Aloinoside B&A				
						Yes	*Heterakis gallinarum*	Mwale et al. (2015)
*Asteraceae*	*Gymnanthemum amygdalinum (Delile) Sch.Bip. (compositae)*	Bitter leaf	Leaves, Roots	Infusion	Tannins, Saponins, alkaloids	No		Jisaka et al. (1992); Alawa et al. (2002); Tuwangye and Olila (2006); Alawa et al. (2010); Nalule and Mbaria (2013); Oyeyemi et al. (2018)
			Leaves			Yes	*Ascaridia galli*	Siamba et al. (2007)
	*Helichrysum splendidum Less.*	Mulberry pines	Flower	Steam Distilled	Phenolics	No		(Mwale and Masika, 2009; Akaberi et al., 2019)
					Flavanoids			
					Terpenes			
					Pyrenes			
					Phloroglucinols			
	*Tanacetum vulgare L.*	Tansy flowers	Flowers	Ethanol extract of dry flowers	Hydroxycinnamic acid, Flavonols, Catechins, tannins, Anthocyans, coumarin	No		(Polovetskaya et al., 2017; Yu et al., 2017)
	*Artemisia cina O.Berg*	Worm seed herb	Flower heads, leaves	Water extraction of dried material	Santonin	No		(Woerdenbag et al., 1997; Valentynivna and Ivanivna, 2017)
					Artemisin			
					Mibulactone			
					Pinene			
	*Artemisia absinthium L.*	Worm wood flower	Leaves, Flowers	Ethyl extraction of dried materials	Absinthin	No		(Patočka & Plucar, 2003; Valentynivna and Ivanivna, 2017)
					Anabsinthin			
					Thujone			
					Monoterpene			
	*Artemisia absinth*	Worm wood flowers				No		Valentynivna and Ivanivna, (2017)
	*Artemisia annua L.*	Wood worm				No		Lans and Turner, (2011)
	*Artemisia absinthium L.*					No		Calzetta et al. (2020)
	*Aretium lappa L.*	Burdock	Fruit	Aqueous ethanolic extracts of the fruit	Arctigenin	No		(Matsumoto et al., 2006; Lans & Turner, 2011)
					Matairesinol			
	*Inula helenium L.*	Elecampane rhizome	Rhizomes and roots	Ethanol extract	Sesquiterpenoid	No		(Valentynivna & Ivanivna, 2017; Das et al., 2020)
					Eudesmanolides			
					Germacranolide			
					Flavonoids			
					Alkaloids			
	*Chamomilla recutita L.*	Chamomile flowers	Flowers	Aqueous and methanolic extracts	Polyphenols	No		(Valentynivna and Ivanivna, 2017; Hajaji et al., 2018)
					Flavonoids			
					Tannins			
	*Cirsium arvense (L.) Scop.*	Canada thistle	Leaves	Methanolic Extracts	Alkaloids	No		(Tulloch and Hoffman, 1982; Lans and Turner, 2011; Banaras et al., 2017)
			Stems		Triterpenes			
			Roots					
	*Taraxacum officinale F.H.Wigg.*	Common dandelion	Roots	Aqueous or Methanol extract	Saponins	No		(Lans and Turner, 2011; Amin Mir et al., 2013)
			Stems		Flavonoids			
			Flowers		Alkaloids			
					Phenols			
*Boraginaceae*	*Symphytum officinale L.*	Comfrey	Roots	Ethanol extract	Alkaloids	No		(Couet et al., 1996; Lans and Turner, 2011; Shang et al., 2018)
			Leaves		Triterpenoids			
					Allantoin			
*Brassicaceae*	*Brassica rapa L.*	Field mustard	Whole plant	Organic solvent extraction, Steaming	Carotenoids	No		(Mahajan and Dua, 1998; Hussain, Khan, Iqbal, and Sajid, 2008)
					Phenolics			
					Progoitrin			
					Flavonoids			
					Phytic acid			
*Bromeliaceae*	*Ananas comosus (L.) Merr.*	Young pineapple	Leaves and Skin	Water extract	Bromelain	No		(Satrija et al., 2001a; Githiori et al., 2004)
*Capparaceae*	*Buchholzia coriacea Engl.*	Wonderful cola	Seed	Ethanol extract	Alkaloids	No		Ibrahim and Fagbohun, (2013); Nweze and Asuzu, 2006)
					Anthraquinones			
					Flavonoids			
					Glycosides			
					Saponins			
					Terpenes			
					Tanins			
*Caprifoliaceae*	*Veleriana officinalis L.*	Velerian rhizomes	Rhizomes and roots	Aqueous ethanol extract	Flavonoids	No		(Valentynivna and Ivanivna, 2017; Nandhini et al., 2018)
					Lignans			
					Valerenic acid			
					Alkaloids			
*Caricaceae*	*Carica papaya L.*	Pawpaw	Seeds	Infusions,	Alkaloids, Proteolytic enzymes, Benzyl isothiocyanate (BITC)	Yes	*Ascaridia galli*	(Stepek et al., 2004; Adu et al., 2009; Nghonjuyi et al., 2020)
			Fruit	Alcohol and water extracts		No		(Satrija et al., 2001a; Chota et al., 2010; Odhong et al., 2014; Ameen et al., 2018)
			Leaves					
			latex			No		(Mursof and Simon, 1991)
*Colchicaceae*	*Gloriosa superba L.*	Flame lily	Tubers, Whole plant	Alcohol extract	Alkaloids	No	*Earthworms*	(Pawar et al., 2010; Suryavanshi et al., 2012; Ashokkumar, 2015)
					Gloriosine			
					Tannins			
					Superbine, Phenols			
*Combretaceae*	*Terminalia avicenniodes Guill.&Perr.*	Terminalia	Roots	Methanol extract	Alkaloids	No		(Ibrahim et al., 1984; Salau et al., 2013)
					Tannins			
					Phlobatannins			
					Phenolics			
					Saponins			
	*Terminalia leiocarpa (DC.)Baill.*	African birch	Leaves, Stem, Root	Aqueous extract	Glycosides	No		(Ibrahim et al., 1984; Mann et al., 2008)
					Phenols			
					Tannins			
					Alkaloids			
					Anthraquinones			
*Cucurbitaceae*	*Momordica chorantia L.*	Bitter melon	Fruit	Alcohol extract	Charantin, Tannins, Phenolics, Terpenoids	Yes	*Ascaridia galli*	(Grover and Yadav, 2004; Alam et al., 2014; Poolperm and Jiraungkoorskul, 2017)
	*Cucurbita Moschanta Duchesne*	Pumpkin	Seeds	Methanol extract of ground seeds	Saponins	Yes	*Ascaridia galli*	(Blancad et al., 1991; Marie-magdeleine et al., 2011)
					Triterpenic compounds			Valentynivna and Ivanivna, (2017)
					Cucurmosin			
					Heterosides			
					Tannins			
	*Cucurbita Moschanta duchesne*	Pumpkin	Seeds	Methanol extract of ground seeds	Cucurbitin	Yes	*Ascaridia galli*	Marie-magdeleine et al. (2011)
					Terpenoids			
					Saponins			
					Sterols			
	*Cucurbita pepo L.*	Summer squash	seeds	Water, ethanol extract	Cucurbitine	No		(AbouLaila et al., 2018; Grzybek et al., 2016)
		Pumpkin			Berberine			
					Palmatine			
					Terpennoid			
					Saponins			
	*Cucurbita maxima Duchesne*	Winter squash	Peels seeds	Alcohol extract	Proteins	No		(Sharma et al., 2013; Chand et al., 2019)
		Pumpkin			Carbohydrates			
					Flavonoids			
					Saponins			
					Tannins			
*Cupressaceae*	*Juniperus communis L.*	Juniper	Fruit	Berry Decoctions	Tannins	No		(Lans and Turner, 2011; Akbar, 2020)
					Diterpenes			
					Biflavonoids			
					Camphene			
*Dryopteridaceae*	*Dryopteris filix-mas (L.) Schott*	Male fern	Leaves, Stems	Ether Extract	Aspidinol	Yes	*Ascaridia galli, Trichostrongylus spp*	(Blakemore et al., 1964; Githiori et al., 2004)
					Flavaspidic acid			
	*Dryopteris inaequalis (Schltdl.) Kuntze*	Ferns	Leaves, rhizomes	Ether Extract	Phloroglucinols	No		(Githiori et al., 2004; Pal Singh and Bharate, (2006)
					Albaspidins			
*Ebenaceae*	*Diospyros mespiliformis Hochst. Ex.A.DC.*	Jackalberry	Roots	Methanol extract	Tannins	No		(Ibrahim et al., 1984; Mammam, 2014)
			Leaves		Saponins			
			Barks		Alkaloids			
					Flavonoids			
*Euphorbiaceae*	*Europhorbia helioscopia L.*	Umbrella milk weed	Stem	Aqueous and Methanol extract	Saponins	No		(Lone et al., 2013; Uzma et al., 2014)
			Leaves		Alkaloids			
			Flowers		Flavonoids			
					Phenols			
	*Mallotus philippinensis (Lam.) Mull.Arg.*	Kamala tree	Fruit	Water or Methanol extract	Phenolics	No		(Akhtar and Ahmad, 1992; Hussain et al., 2008)
					Flavones			
					Saponins			
					Tannins			
					Triterpenes			
	*Codiaceum variagetum (L.) Rumph. Ex A.Juss.*	Croton	Leaves	Ethanol, Water extracts	Phenolics	No		(Satrija et al., 2001b; Mohamed et al., 2019)
					Flavonoids			
					Alkaloids			
					Saponins			
					Terpenoids			
					Tannins			
*Fabaceae*	*Senna occidentalis (L.)*	Coffee Senna	Stem barks	Cold methanol extraction	Glycosides	No		(Suleiman et al., 2014; Raza et al., 2016)
					Tannins			
					Flavonoids			
					Saponins			
					Triterpenes			
	*Tephrosia villosa (L.) Pers.*	Hoary Tephrosia	Leaves Stems	Methanolic extract	Polyphenols	No		Ahmad and Khan, (2013); Odhong et al., 2014)
					Tannins			
					Alkaloids			
					Anthocyanins			
					Rotenoids			
	*Milletia grandis (E.Mey.)Skeels*	Umzimbeet	Leaves	Cold water extraction	Not Analysed	No		Mwale and Masika, (2015)
	*Trifolium repens L.*	White clover	Aerial shoot	Methanol extract	Flavonoids	No	*Earthworms*	(Tangpu et al., 2004; Sabudak and Guler, 2009)
					Isoflavonoids			
					Chalcones			
					Coumarins			
	*Flemingia vestita L.*	Sohphlang	Root tuber	Spirit extract of dried root peels	Genistein	Yes	*Earthworm*	(Tandon et al., 1997; Shailajan et al., 2014)
							*Ascaridia galli*	
							*Heterakis gallinarum*	
	*Sesbania grandiflora (L.) Poir.*	Vegetable hummingbird	Flowers	Water extracts	Saponins	Yes	*Ascaridia galli*	(Sable and Dhawale, 2013; Karumari et al., 2014)
					Proteins			
					Flavonoids			
					Alkaloids			
					Tannins			
	*Leucaena leucophala (Lam.) de Wit*	Ipil-ipil	Seed Leaves	Water extract of dried materials	Quercetin	No		(Satrija et al., 2001a; Ozaraga et al. (2017)
					Caffeic acid			
	*Senegalia mellifera (Benth.) Seigler &Ebinger*	Blackthorn	Stem barks	Methanol extracts	Triterpenoids	No		(Githiori et al., 2004; Mutai et al., 2004)
					Lupenone			
					Betulin			
					Alkaloids			
	*Senna occidentalis (L.)*	Coffee senna	Stem barks	Methanol extracts	Flavonoids	Yes	*Heterakis gallinarum, Ascaridia galli*	(Kateregga et al., 2014; Suleiman et al., 2014)
					Tannins			
					Alkaloids			
					Triterpenes			
					Anthraquinones			
	*Parkia platycephala*	African locust bean	Leaves Seeds	Acetone-water extract	Phenols	No		Oliveira et al. (2017)
					Flavones			
					Phytosteroids			
					Tannins			
	*Dimorphandra gardneriana Tul.*	Fava d’anta	Leaves, bark	Acetone-water extract	Flavonoids	No		Oliveira et al. (2017)
					Phenols			
					Tannins			
					Saponins			
	*Vachellia nilotica (L.) P.J.H.Hurter&Mabb.*	Thorn mimosa	Fruit Bark	Methanolic extracts	Tannins	No		(Bachaya, Iqbal, Khan, Sindhu, and Jabbar, 2009; Banso, 2009)
					Terpenoids			
					Saponins			
*Gunneraceae*	*Gunnera perpensa L.*	River pumpkin	Leaves	Water extract	Alkaloids	Yes	*Heterakis gallinarum*	(Mwale and Masika, 2015; Maroyi, 2016)
					Benziquinones			
					Ellagic acids			
					Flavonoids			
					Phenols			
					Proanthocyanidins, tannins			
*Lamiaceae*	*Mentha x piperita L.*	Peppermint	Leaves	Hot water extract	Β-sitosterol	No		(Githiori et al., 2005; Bartolome et al., 2013)
	*Clerodendum colebrookianum Walp.*	East Indian glory bower	Stalk Leaves	Methanolic extracts	Phenolics	No	*Earthworm*	(Tandon et al., 2011; Yadav and Temjenmongla, 2012; Das et al., 2013)
					Flavonoids			
					Carbohydrates			
					Alkaloids			
					Tannins			
					Gallic acid			
	*Coleus scutellarioides (L.) Benth.*	Coleus	Leaves	Juice of leaves	Flavonoids	Yes	*Chicken tapeworm*	Satrija et al. (2001b)
					Tannins			
					Saponins			
	*Mentha longifolia (L.) L.*	Wild mint	Leaves	Aqueous and HCL extract	Piperitenone oxide	Yes	*Ascaridia galli*	(Ghoulami et al., 2001; Ali et al., 2013)
					Piiperitone oxide			
	*Mentha x piperata L.*	Peppermint	Stems, leaves and roots	Methanol extraction of dry material	Menthone	No		(Girme et al., 1970; Lans & Turner, 2011; Freire et al., 2012)
					Neomenthol			
					Menthol			
					Carvone			
*Leguminosae*	*Tephrosia vogelli Hook.f.*	Fish poison bean	Leaves Stems	Soxhlet method, maceration of ethanolic extracts.	Polyphenols	Yes	*Ascaridia galli*	(Siamba et al., 2007; Kabera et al., 2014)
					Tannins			
					Alkaloids			
					Anthocyanins			
					Rotenoids			
	*Albizia antihelmintica (A.Rich.) Brongn.*	Worm cure Albizia	Stem barks leaves	Drying and pounding, Methanol extract	Flavonoids	No		(Githiori et al., 2004; Gradé et al., 2008)
					Galloyl glucosides			
					Piscidic acid			
*Lythraceae*	*Punica granatum L.*	Pomegranate	Peels	Methanol extract	Gallotannins	Yes	*Ascaridia galli*	Aziz et al. (2018); Madrigal-Carballo et al., 2009)
					Ellagitannins			
					Anthrocyanins			
					Polyphenols			
					Tannins			
*Malvaceae*	*Gomphocarpus fruticosus (L.) W.T.Aiton*	Cotton bush	Leaves	Ethanolic extract	Tannins	No	*Earthworms*	Ade-Ademilua and Okpoma, 2018; Lans and Turner, 2011; Nandeeshwar et al., 2019
					Phenols			
					Flavonoids			
*Meliaceae*	*Azadirachta indica A.Juss.*	Neem	Leaves	Pound and mixed with feeds	Alkaloids	Yes	*Ascaridia galli*	Pande and Tiwari, (2007); Susmitha et al., 2013)
			Stem barks		Glycosides			
					Terpenoids			
					Tannins			
					Flavonoids,			
					Sugars			
				Ethanol, Water extract		Yes	*Ascaridia galli*	Alam et al. (2014)
*Mimosaceae*	*Mimosa pudica L.*	Shame plant	Leaves	Ethanol extract	Alkaloids	Yes	*Ascaridia galli*	(Nghonjuyi et al., 2020; Tamilarasi and Ananthi, (2012)
					Steroids			
					Flavonoids			
					Phenols			
*Moraceae*	*Ficus sycomorus L.*	Sycamore fig	Stem barks	Aqueous extract	Polyuronides	No		(Githiori et al., 2005; Sandabe et al., 2006; Piña-Vázquez et al., 2017)
					Gallic acid			
					Catechol tannins			
					Saponin			
					Alkaloids	
*Myrtaceae*	*Psidium guajava L.*	Common guava	Leaves	Water extraction	Limonene	No	*Earthworm*	(Piña-Vázquez et al., 2017)(Jaiarj et al., 1999; Ogunwande et al., 2003; Tandon, et al., 2011)
					b-caryophyllene			
					b-bisabolene			
*Papaveraceae*	*Papaver somniferum L.*	Opium poppy	Leaves	Ethanol extract	Morphine	No		(Lans and Turner, 2011; Masihuddin et al., 2018)
			Fruits		Isoquonolones			
			Seeds					
			Latex					
*Passifloraceae*	*Turnera ulmifolia L.*	Yellow alder	Leaves Roots	Hydroalcoholic extract	Phenols	No		Oliveira et al. (2017)
					Tannins			
					Cumarins			
					Saponins			
*Piperaceae*	*Piper betle L.*	Betle leaf	Stems	Ethanolic extracts		No		(Adate et al., 2012; Raza et al., 2016)
*Rhaminaceae*	*Frangula pushiana (DC.) A. Gray ex J.G. Cooper*	Buckthorn bark	Stem barks	Aqueous ethanol extract	Sesquiterpenoids	No		Valentynivna & Ivanivna, (2017)
					Flavonoids			
					Tannins			
					Steroids			
	*Ziziphus nummularia (Burm.f.) Wight&Am.*	Lotebush	Bark	Methanolic extract	Phenols	No		Bachaya et al. (2009)
					Flavonoids			
					Alkaloids			
					Saponins			
*Rosaceae*	*Rubus fruticosus L.*	Black berry	Leaves Fruits	Methanol extract	Tannins	Yes	*Ascaridia galli*	(Lans and Turner, 2011; Ali et al., 2013)
					Flavonoids			
					Sesquiterpenes			
					Saponins			
*Rubiaceae*	*Morinda citrifolia L.*	Indian mulberry	Leaves	Alcohol extract	Carbohydrates	No		(Hirazumi and Furusawa, 1999; Raza et al., 2016)
					Arabinogalactan-proteins			
					Phenolics			
*Rutaceae*	*Tetradium rutaecarpa (A.Juss.) T.g.Hartley*	Medicinal evodia	Fruit	Methanol- fruit extract	Atanine	No		(Githiori et al., 2005; Lian et al., 2020)
*Sapotaceae*	*Madhuca longifolia var. latifolia (Roxb.) A.Chev.*	Butter tree	Seeds	Water extracts	Tannins, Sugars,gallic Phenolics, flavanols, Catechins	No		Asadullar and Sabir, (1980); Raza et al., 2016)
*Saururaceae*	*Honttuynia cordata Thunb.*	Chameleon plant	Leaves	Dry leaf water extract	B-myrcene	No	*Earthworms*	(Dai et al., 2015; Tandon, et al., 2011)
					Monoterpene			
					Aliphatic ketones			
*Solanaceae*	*Solanum torvum Sw.*	Turkey berry	Fruit Leaves	Water extract of powdered fruits or leaves	Flavonoids	Yes	*Ascaridia galli*	Karumari et al. (2014)
					Alkaloids			
					Phenols			
					Tannins			
					Saponins			
	*Nicotiana tabacum L.*	Tobacco	Leaves	Aqueous, Methanol extracts	Alkaloids	No		Lans and Turner, (2011)
*Verbenaceae*	*Duranta erecta L.*	Golden dewdrop	Fruit	Methanolic extract	Flavonoids	No		(Udobi et al., 2018; Calzetta et al., 2020)
					Tannins			
					Terpenes			
					Polyuronides			
					Saponis			
*Zygophyllaceae*	*Tribulus terrestris L.*	Puncture vine)	Whole plant	Methanol extract	Saponins, Tribulosin, B-sitosterol-D-glucoside	No		(Deepak et al., 2010; Chhatre et al., 2014; Raza et al., 2016)

Synthetic anthelmintics leave residue in poultry products, residues which have been associated with carcinogenesis and anthelmintic resistance (Patel et al., 2018). The synthetic anthelmintics are not only expensive but also reduce the acceptability of poultry products (Hammond et al., 1997). These issues are of particular concern especially in low and middle-income countries where weak legislation and infrastructure for monitoring anthelmintics in animal products continues to prevail. Investing capital to promote research towards the promotion of reliable and tested plant based products would be of benefit to farmers and could provide novel alternatives for the treatment of emerging anthelmintic resistant strains in poultry. This is important since such plants can easily be grown for farm (plant medicines are acceptable in organic farming) or industrial use. However, the concentration of the phyto-compounds varies with the seasons and locations. Some plants are only found in particular parts of the world and may not even grow in others. The farm methods of using plant anthelmintics are more likely to cause toxic effects than the industrial laboratory methods in the absence of clear clinical data to guide their adoption (Hammond et al., 1997).

### 3.2 The Demand for Alternative Anthelmintic Options for Poultry Production

Intestinal helminths infections are a major cause for concern (Bazh and El-bahy, 2013). While helminths have many different predilection sites (Table 1), helminth species in poultry are characterized by their hepato-pulmonary migration with an escape to the peritoneum which results in abdominal peritonitis and intestinal perforation (Bazh and El-bahy, 2013). *Ascaridia galli, Heterakis gallinarum,* and *Capillaria spp*. penetrate the mucosa causing hemorrhage and subsequently return to the lumen to reach maturity and this infection has been associated with reduced weight gain and productivity losses (Bessell et al., 2012; Collins et al., 2015). The lack of a humoral immune response (Thapa et al., 2018) against helminths has led to dependence on anthelmintics and the search for more potent chemotherapeutical agents.

Benzimidazoles (BMZ) are commonly used in poultry including flubendazole, fenbendazole, and albendazole. Flimabend^®^ (flubendazole) is used to treat *Ascaridia galli*, *Heterakis gallinarum*, and *Capillaria* spp. Flubendazole ([5-(4-fluorobenzoyl)-1H-benzimidazole-2-y1]-carbamic acid methyl ester) a benzimidazole carbamate (Levkut et al., 2017) impairs tubulin polymerization into microtubules, contributing to death of parasite (Zamanian et al., 2018). These drugs are applied daily to achieve therapeutical effects but increasing drug resistance leads to rapid reinfection, inadequate drug delivery, and increased farm losses through increased production costs (Collins et al., 2015). Overuse of anthelmintics including levamisole subsequently leads to drug residues in poultry products (Bilandz et al., 2018). In humans, consuming such products leads to hypersensitivity reactions including nausea, gastrointestinal manifestations, fever, and neurological effects (Levkut et al., 2017).

The lack of scientific studies on alternative anthelmintic options undermines efforts for alternative medicinal options (Bazh & El-bahy, 2013; Rana & Misra-Bhattacharya, 2013). Limited availability and high cost of synthetic anthelmintics have generated an increased interest in ethnoveterinary medicines and the search for new plant compounds for helminth control (Rana & Misra-Bhattacharya, 2013; Scantlebury et al., 2013). There is a growing movement in some low and middle-income countries to recognize the value of traditional medicinal approaches. There is a need to document the use of anthelmintic agents in communities as oral traditions in local medicine systems are fragile and there is a real risk of loss (Mcgaw et al., 2020).

Plant products are used in various regions for the treatment of poultry diseases. Farmatan® is a natural extract derived from chestnut wood (*Castanea sativa miller*), from the Fabaceae family. The primary component is water-soluble vegetable polyphenols—tannins that impairs nematode larval development and viability (Levkut et al., 2017). Ethnoveterinary medicine offers a cheaper and accessible option because products are locally available (Nghonjuyi et al., 2016). Plant compounds vary in quality and quantity with the different geographical zones; season of the year, nature of the solvent used, and particular edaphic factors. Not all compounds have been tested on synthetic anthelmintic resistant helminths but some terpenoids were effective against albendazole resistant helminths (Mirza et al., 2020). The details that make particular plant compounds effective against helminths resistant to synthetic anthelmintics are not yet known. There are several plant candidates whose compounds have been proven to be effective against helminths in animal species other than poultry, those have been listed in Table 2 as possible opportunities. However, the lack of information on efficacy, standardizations, and toxicity of the plant compounds in animals like chickens continues to challenge possible ratification (Mcgaw et al., 2020).

### 3.3 Ethnoveterinary Medicine in Veterinary Practice

Ethnoveterinary practices are commonly used to target poultry helminths. A pool of plants and practices have passed through generations that are used to manage poultry diseases (Gueye, 1999; Mwale et al., 2005). Usually, one herb is said to be effective on a variety of diseases including helminthiasis (Dhama et al., 2015). The value of such products is sometimes doubted because of a lack of scientific proof (Toyang et al., 1995). Practices are passed from one generation to the next through informal classes and storytelling. This inadvertent research on ethnoveterinary medicine has acceptance because it is tailored to the cultures and traditions of various communities. While plants used tend to be available locally, extraction is challenging and can affect the result. Polyphenolic compounds of *Rubus ulmifolius schott* varied with the varying polarity and quality of solvents (Dev et al., 2015; Akkari et al., 2016) Even the efficacy of extracts depends on the solvent used (Wang, 2011; Akkari et al., 2016). Helminths are possibly the most important poultry parasites (Ruff, 1999) and ethnoveterinary practices may offer alternative products to combat anthelmintic resistance.

### 3.4 Plant Metabolites

#### 3.4.1 Phenolics

**FIGURE 1:**
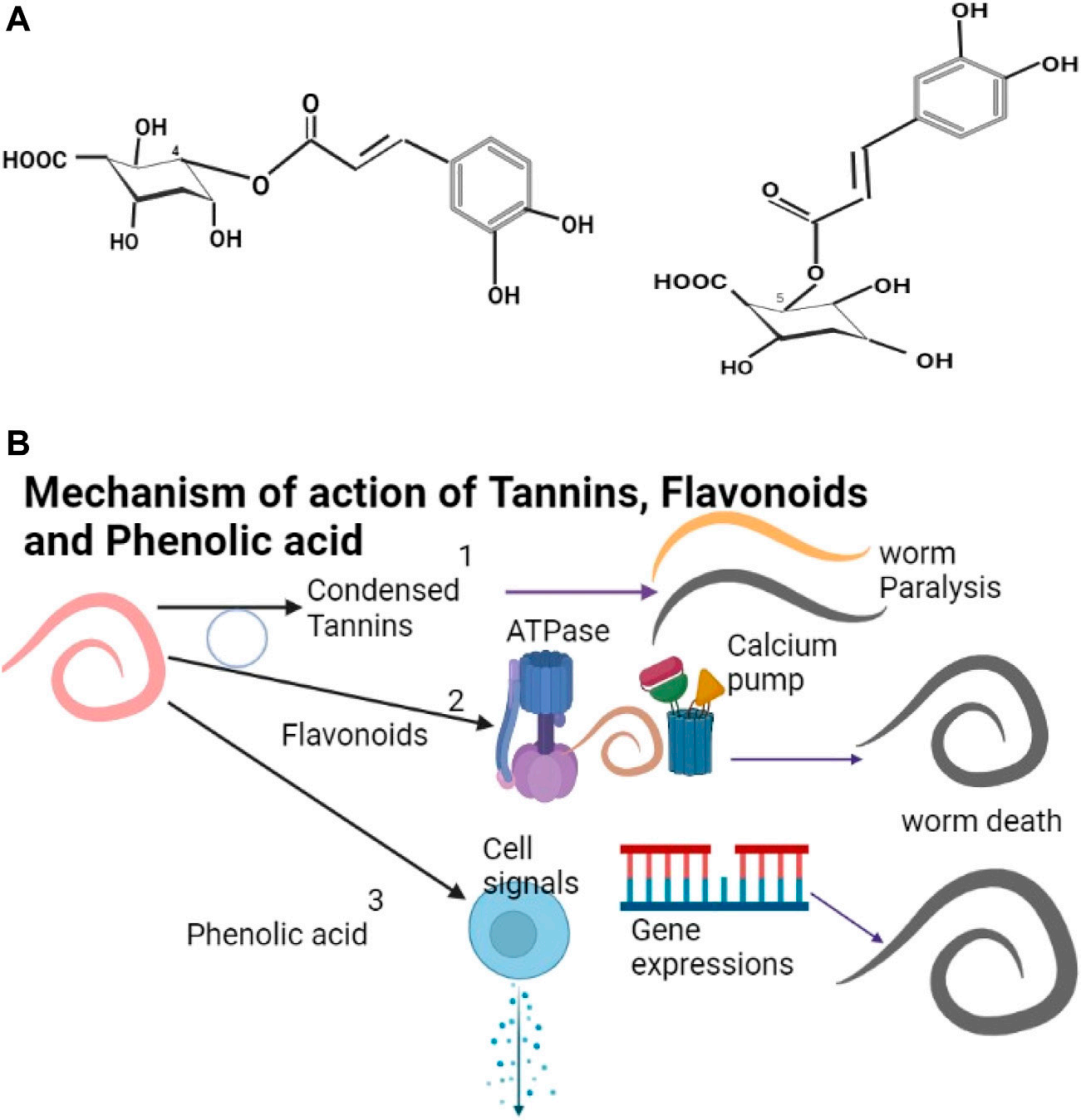
**(A)** 4-O-Caffeoylquinic acid and 5-O-caffeoylquinic acids are examples of phenolic acids in green coffee beans (Wei and Tanokura, 2015). **(B)** (1) Condensed Tannins cause paralysis and death of helminths. (2) Flavonoids affect the calcium pump and ATPase leading to the death of the helminth. (3) Phenolic acids affect cell signaling pathways and gene expressions leading to the death of the helminth. The blue circle shows possible synergistic actions between Condensed Tannins and Flavonoids.

The phenolics include tannins, flavonoids, and phenolic acids (example of structures shown as Figure 1A) with catechins, anthocyanins, and coumarins being derivatives of phenolics (Dai and Mumper, 2010; Shen et al., 2017). Tannins are plant polyphenols (Salminen et al., 2011) with a similar structure to that of synthetic phenols. Tannins are found in many families of plants including Asteraceae, Anacardiaceae, Leguminoseae, Lumiaceae, Apiaceae and Cucurbitaceae (Table 2). Catechins and epicatechins are monomers that make the tannins (Duval and Avérous, 2016). Tannins can only be degraded at very high temperatures (190°C) and can be processed at high temperatures (García et al., 2014). Condensed tannins (CT) from dicotyledonous plants (Kahn and Diaz-Hernandez, 1999) are the major metabolites that cause the plant anthelmintic properties (Anthanasiadou et al., 2001; Patilaya et al., 2017). Although tannins have beneficial effects they require to be administered with care (Marzoni et al., 2020) due to their anti-nutritional character (Mansoori and Modirsanei, 2012). They are effective anthelmintic agents (Cenci et al., 2007; Sandoval-Castro et al., 2012) and cause nematode paralysis and death (Figure 1B), leading to expulsion from the gastro-intestinal tract (Kane et al., 2009). Controlled feeding of animals on tannins has many advantages (Kabasa et al., 2000; Alonso-Díaz et al., 2010).

Flavonoids are polyphenolic compounds (Cook and Samman, 1996; Croft, 1998; Madhusudhana et al., 2010) for which there is no database on the physicochemical properties (Kinoshita et al., 2005). Flavonoids may act by the various enzymes (Figure 1B) like phosphodiesterase and Ca2+-ATPase (Cazarolli et al., 2008; Rathee et al., 2009) but little is known as to their mode of action (Kerboeuf et al., 2008). Flavonoids have been identified in the families Rosaceae, Solanaceae, Fabaceae, Anacardiaceae, Euphorbiaceae, and Brassicaceae (Table 2). Flavonoids are effective against helminths (Pereira et al., 2016; Fomum and Nsahlai, 2017) and act synergistically with tannins (Klongsiriwet et al., 2015); they also potentiate the action of praziquantel against helminths (Hrckova & Velebny, 2010). Catechins can form tannin substances (Smeriglio, Barreca, Bellocco, and Trombetta, 2016) but are unstable and have limited application (Castañeda-Ovando et al., 2009; Dube et al., 2010). Catechins can be stabilized by encapsulation in B-cyclodextrin (Ho et al., 2017) or chitosan-tripolyphosphate (Dube et al., 2010) and their release from complexes increases with an increase in temperature (Bian et al., 2019). Coumarins also have anthelmintic effects (Kamble et al., 2013; Torres et al., 2014) and can be easily transformed into various useful derivatives (Katsori and Hadjipavlou-Litina, 2014; Stefanachi et al., 2018). Phenolic acids have been found in some members of the plant families of Colchicaceae, Solanaceae, Cucurbitaceae, Euphorbiaceae, Malyaceae and Brassicaceae and show anthelmintic properties (Akter et al., 2014; Ndhlala et al., 2015) (Table 2). Phenolics affect cell signaling pathways and gene expression (Figure 1B).

#### 3.4.2 Alkaloids

**FIGURE 2:**
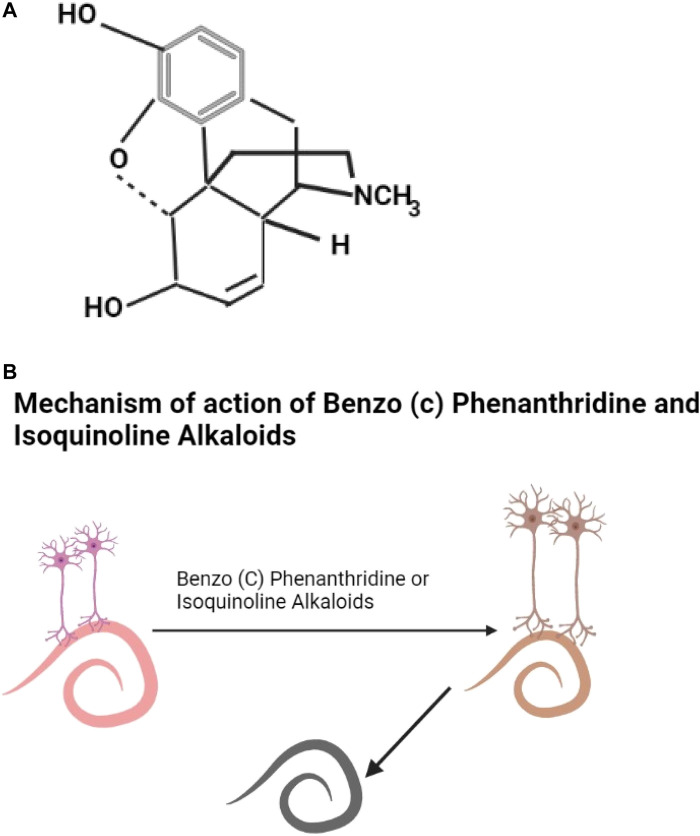
**(A)** The structure of morphine (Verpoorte, 2005). **(B)** Benzo (c) phenanthridine or isoquinoline alkaloids damage helminth neurons leading to the death of the helminth.

Alkaloids are secondary metabolites, this group includes morphine, quinine, strychnine, atropine, colchine, and nicotine (Gutiérrez-Grijalva et al., 2020). An example of an alkaloid structure is shown as Figure 2A. They are most common in herbaceous plants (Jirschitzka et al., 2013; Fester, 2018). Alkaloids are found in abundance the Solanaceae and Erythroxylaceae plant families (Kohnen-Johannsen and Kayser, 2019) but are also found in some plants in the families of Leguminoseae, Meliaceae, Caprifoliaceae, and Euphorbiaceae. They are effective against helminths (Perrett and Whitefield, 1995; Ayers et al., 2007; Rocha et al., 2017) though toxic (Moreira et al., 2018); they show promising neurotoxic pharmacological properties in helminths (Terada et al., 1982; Athanasiadou et al., 2007) (Figure 2B) (Table 2).

#### 3.4.3 Saponins

**FIGURE 3:**
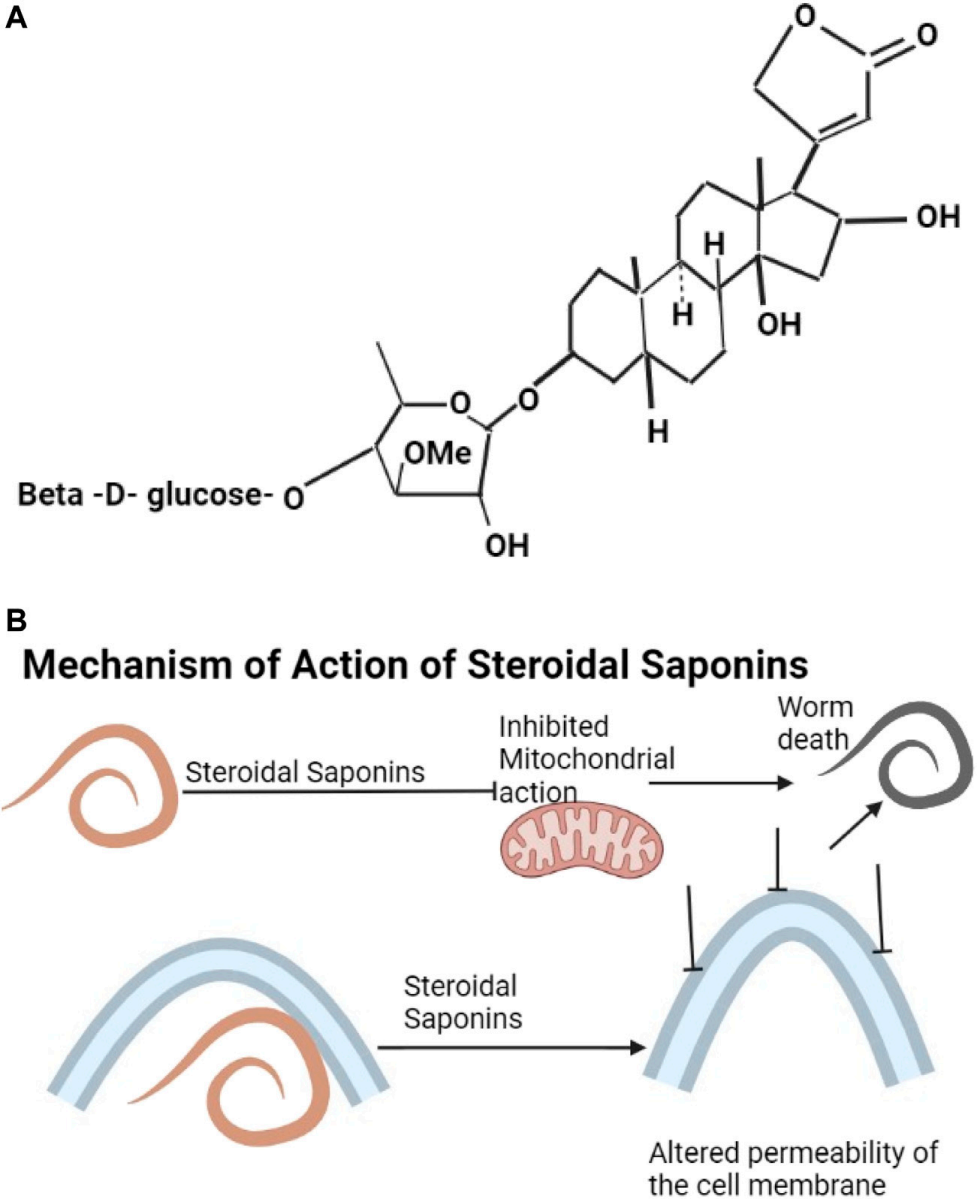
**(A)** The structure of digitalin is an example of steroidal saponins (Morgan and Wilson, 1999). **(B)** Saponins affect mitochondrial action and also alter the permeability of the cell membrane leading to death of the helminth.

Saponins include triterpenoids, steroids, gensenosides, allium saponins, glycoalkaloid saponins (Savage et al., 2003; Sobolewska et al., 2016). The structure of digitalin which is a steroidal saponin is shown as Figure 3A. Plant saponins are found in plants of the families of Fabaceae, Zygophyllaceae, Rosaceae, Apiaceae, and Verbenaceae (Table 2). They contain triterpene and sugar chains of varying lengths (Guclu-Ustundag and Mazza, 2007) and have the properties of foaming, solubilization, and emulsification (Ribeiro et al., 2013). Saponins are amphiphilic (Singh and Chaudhuri, 2018) and interact with sterols to form a variety of biological compounds of various categories of pharmaceutical values (Ribeiro et al., 2013). Ginseng plants are rich sources of saponins (Shi et al., 2019; Wang et al., 2020). Saponins have anthelmintic properties (Wang et al., 2010; Idris et al., 2017) affecting mitochondrial action (Santos et al., 2018) and altering the permeability of the helminth cell membrane, leading to damage of the helminth (Melzig et al., 2001) (Figure 3B).

#### 3.4.4 Terpenoids

**FIGURE 4:**
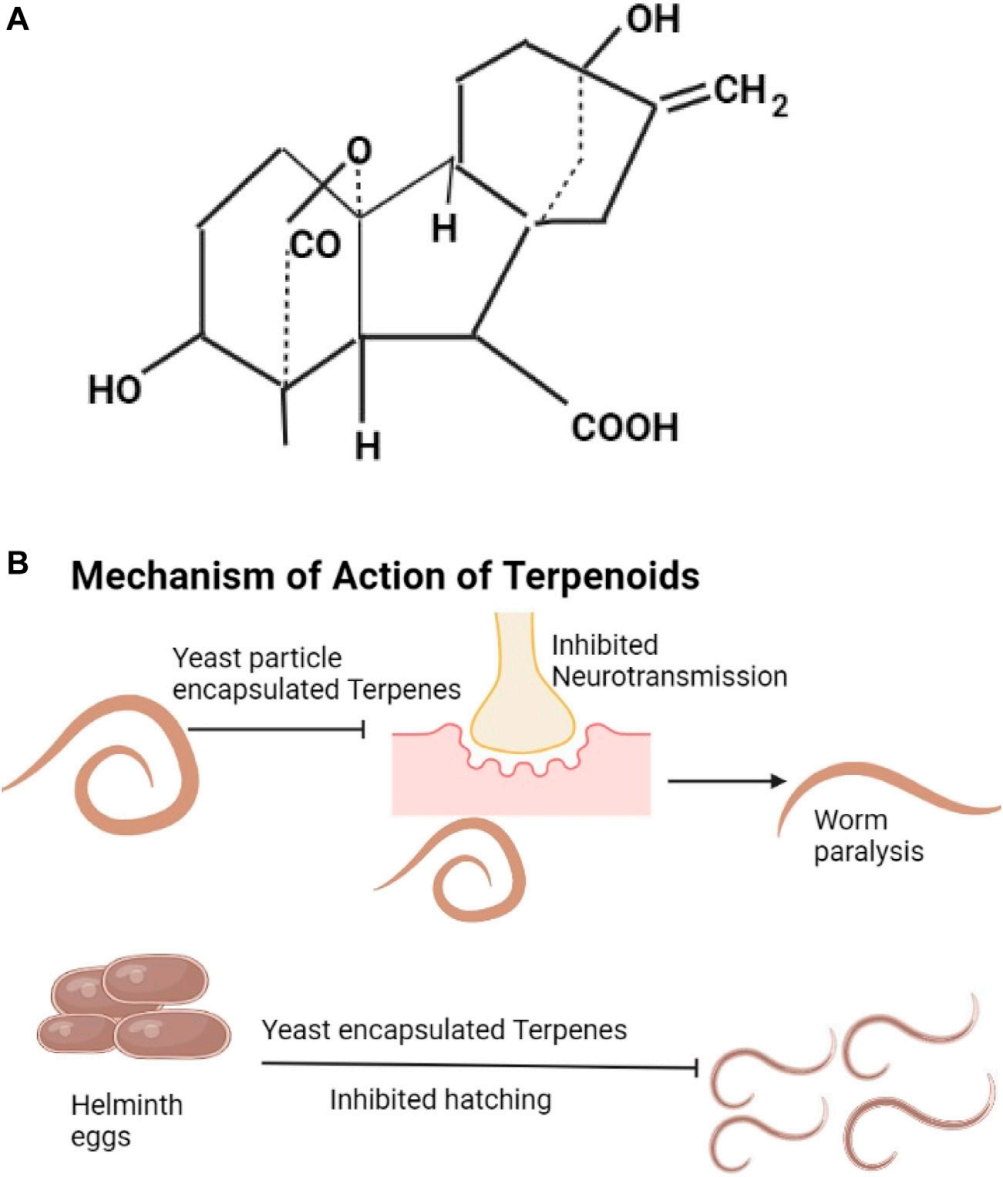
**(A)** The structure of gibberellic acid (Sponsel, 2003). **(B)** Yeast encapsulated terpenes inhibit neurotransmission and lead to helminth paralysis, they also inhibit hatching of helminths eggs.

Digoxin, cicutoxin, atractyloside, daphetoxin, gibberellic acid (The example given as Figure 4A), betulinic acid, lupeol, ursolic acid, and oleanolic acid are the different types of terpenoids. Digoxin is from plants like *Digitalis lanata*, it affects muscle contraction through increasing calcium ions (Joel Edwards, 2009). Atractyloside is found in *Callilepsis laureola* and *Atractylis glummifera*, it blocks oxidative phosphorylation (Mbaveng et al., 2014). Gibberellic acids are found in *Capsicum annuum*, they decrease anti-oxidant enzymes (Mbaveng et al., 2014). Diphnetoxins are found in plants of the family Thymelaeaceae, they inhibit ATP synthase and the mitochondrial respiratory chain (Diogo et al., 2009). Betulinic acids are found in the bark of various plants, information about their actions is not available, they are thought to increase cytochrome C release by acting on the mitochondria (Varsha et al., 2017). Lupeol is from the bark of *Bombax ceiba* and *Albizia adianthifolia* (Fabaceae), lupeol suppresses the cells of inflammation (Dev et al., 2017; Saleem, 2009). Oleanolic acids are from fruits and vegetables especially *Olea europaea*, they lower glucose levels by mechanisms that are not well understood (Rodriguez-Rodriguez and Ruiz-Gutierrez, 2010; Yagishita et al., 2016). Ursolic acids are found in bilberries, apple peels, and peppermint, they cause increased Akt activity and increased energy utilization (Baliga et al., 2019; Wicks et al., 2018). Terpenoids with known anthelmintic properties include menthol and camphor (Mukherjee et al., 2016). Terpenoids hinder neurotransmission leading to helminth paralysis and inhibition of worm egg hatching (Mukherjee et al., 2016; Pillai & Nair, 2011) (Figure 4B). Terpenes have been shown to control albendazole-resistant helminths (Mirza et al., 2020). The specific properties that make particular terpenes effective against albendazole-resistant helminths are not known.

#### 3.4.5 Glucosinolates

**FIGURE 5:**
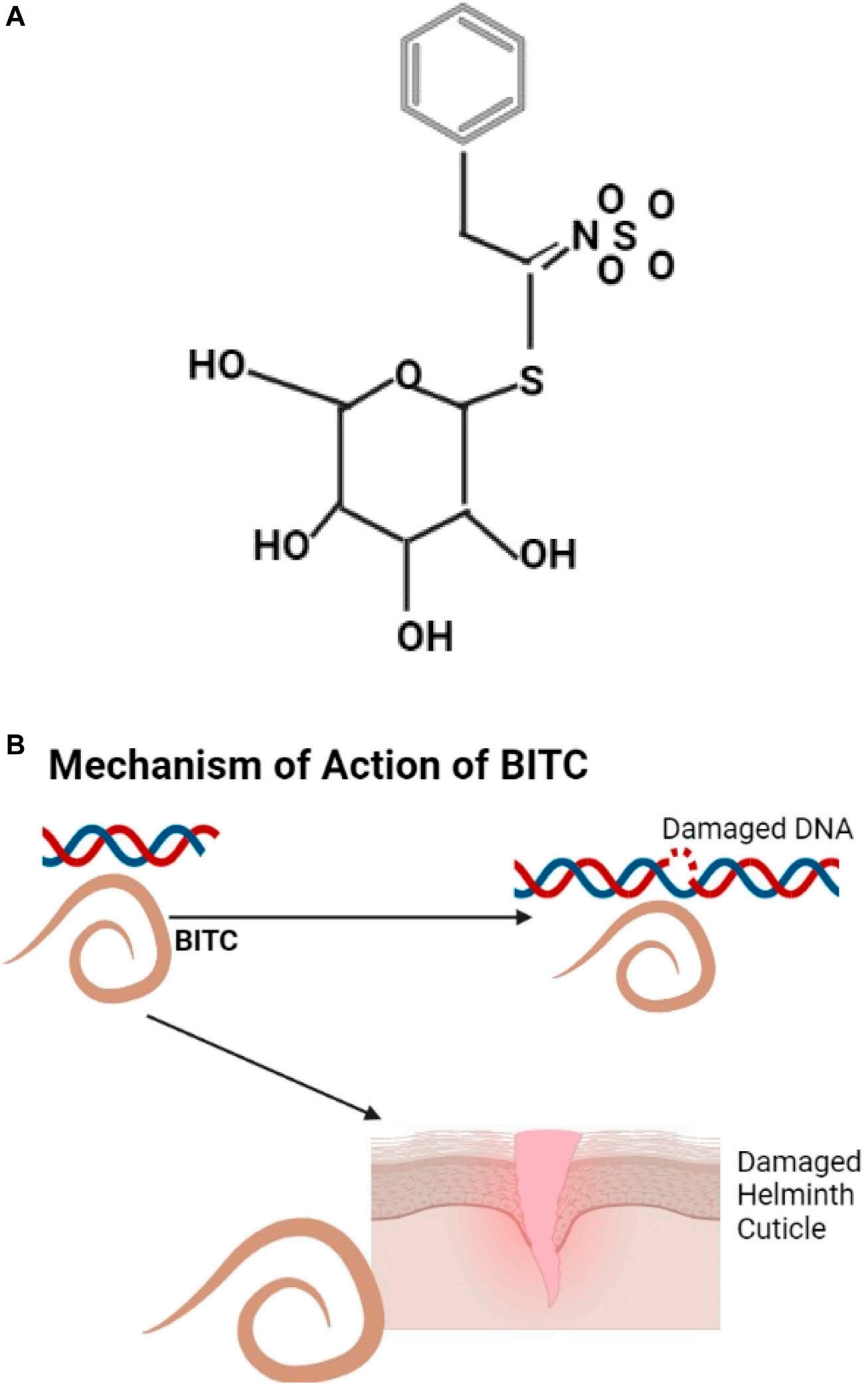
**(A)** The structure of Benzyl glucosinolate, metabolites of glucosinolates (Akram et al., 2021). **(B)** BITC causes helminth DNA and cuticle damage.

An example of glucosinolate is shown (Figure 5A), they are natural glucosides that can be aliphatic, indole, and aromatic (Ishida et al., 2014). Annonaceae (Papaya) has anthelmintic properties; the papaya seeds have benzyl glucosinolate that is hydrolyzed by the enzyme myrosinase to benzyl Isothiocyanate (BITC) (Wilson et al., 2002) known to be effective against helminths (Kermanshai et al., 2001). BITC is thought to act by protein modification (Goosen et al., 2000) or by causing DNA damage (Kassie et al., 1999) (Figure 5B). Cysteine proteinases from papaya include Papain and chymopapain which digest the helminth cuticle (Stepek et al., 2004; Behnke et al., 2008).

#### 3.4.6 Isoflavones

**FIGURE 6:**
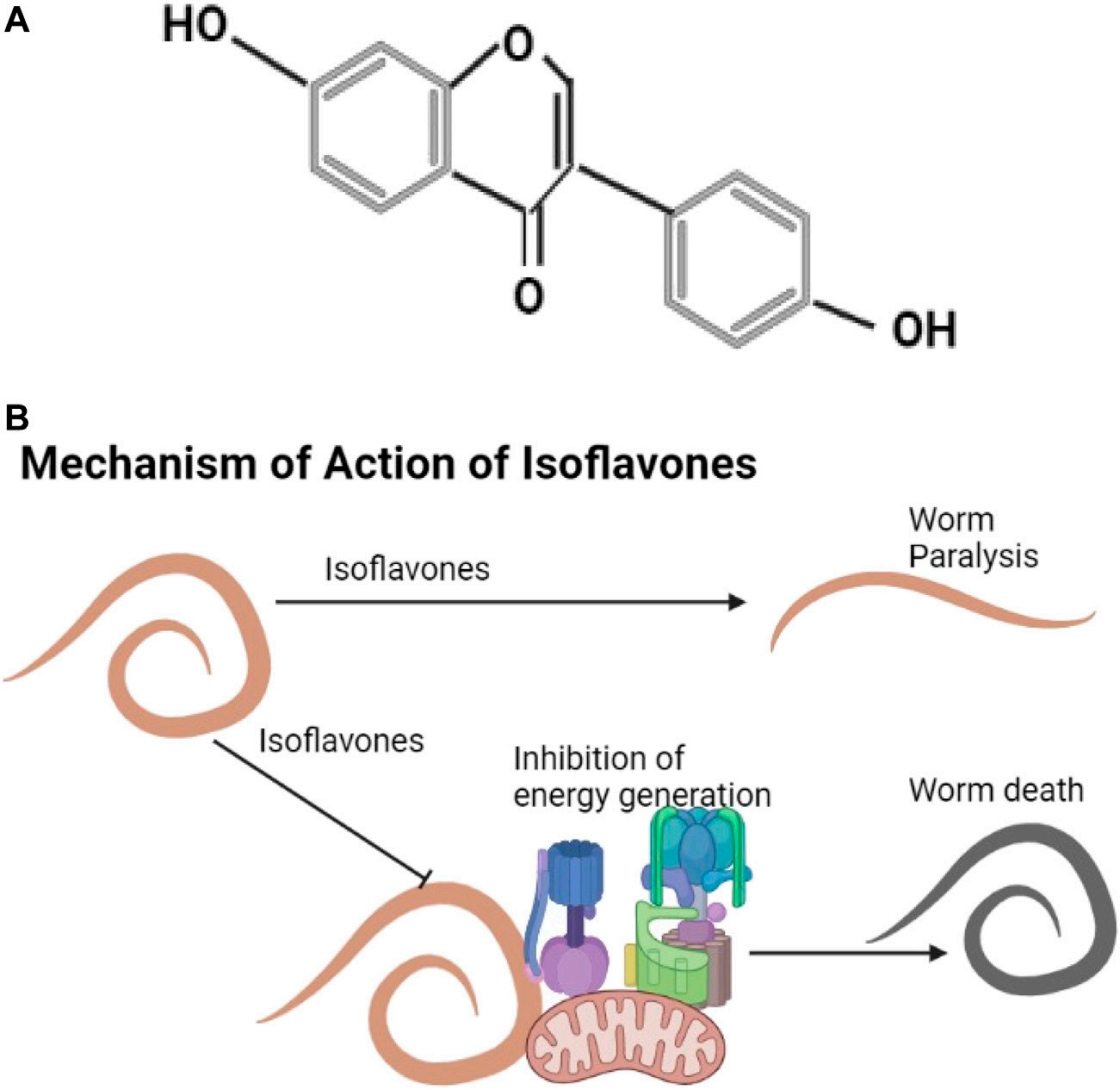
**(A)** The structure of daidzein (Hampl et al., 2009). **(B)** Isoflavones cause helminth paralysis and inhibit energy utilization.

Daidzein shown is an example of an isoflavone (Figure 6A). They are found in plants like *Trifolium subterraneum*, *Medicago spp.* (Barreira et al., 2015) and in soya agricultural waste (Carneiro et al., 2020). Known isoflavones include genistein, formononetin, pseudobaptigenin, and daidzein that cause helminth flaccid paralysis, inhibit energy generation, and affect calcium utilization (Das et al., 2006; Nirala et al., 2019) (Figure 6B).

#### 3.4.7 Artemisinin and Its Derivatives

**FIGURE 7:**
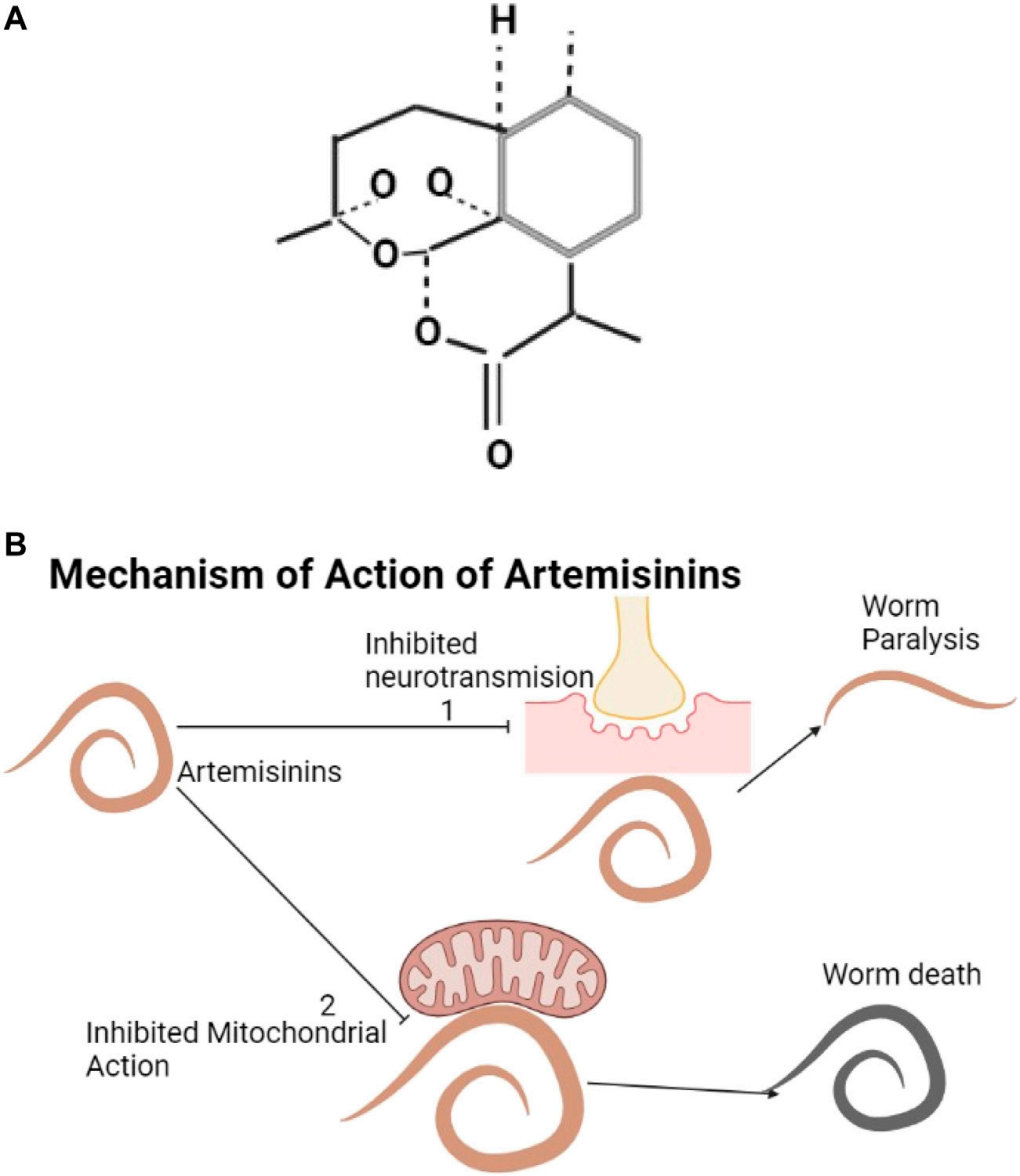
**(A)** The structure of artemisinin (Zeyuan, Yulin, and Meiyi, 2018). **(B)** Artemisinin and its derivatives inhibit neurotransmission resulting into worm paralysis (1) and can affect mitochondrial action resulting into worm death (2).

The structure of artemisinin is shown below (Figure 7A). Artemisinin and its derivatives are found in *Artemisia annua*. They produce oxygen radicals and can influence inhibitory neurotransmission (Pacios-Michelena et al., 2021). These cause oxidative stress through the effects on mitochondrial action and electron transfer in the parasite (Cumming et al., 1998; Beshay, 2018) and kill helminths (Fathy, 2011; Cala et al., 2014) (Figure 7B).

### 3.5 Relevance of the Review to Drug Policy and Planning

The efficacy of synthetic chemical anthelmintics is challenged by the escalating anthelmintic resistance and ethno-compounds are a growing resilient substitute due to their ecological stability. Resource-poor farmers can be helped to develop cheap plant resources which are sustainable and easy to process. Anthelmintic ethno-compounds can be a basis for nano-anthelmintics or green synthesis for possibly better-performing anthelmintics. Silver nanoparticles have been shown to potentiate the action of *Momordica charantia L.* fruit extract against helminths (Rashid et al., 2016) and gold nanoparticles make the fungus *Nigrospora oryzae* more effective against poultry tapeworms (*Raillietina spp*) (Kar et al., 2014).

While in China and the Himalayan regions ethnobotany has guided the new drug development (Sheng-Ji, 2001) this has not occurred in many low-income countries. The People’s Republic of China has developed ethnopharmacology alongside Western medicine by the establishment of a robust ethnomedicinal databank and funding research in this field. In comparison, developing countries (especially in Africa) lack clear policies on community-based research and sustainable ethnoveterinary resource management since legislation is crucial for national and international adoption of ethnomedicines.

A lack of poultry drug use policy in developing countries makes farmers vulnerable to unscrupulous dealers (unlicensed drug dealers) who exploit farmers with fake products for the sake of money. Farmers may buy useless products with the hope of treating their flocks and end up losing their flocks. For example, the use of opium poppy (*Papaver somniferum L.*) in control of poultry helminths is very common (Lans and Turner, 2011) but some low-income countries lack clear regulations and policies on the medicinal use of narcotic plants. There are no frameworks of access and benefit-sharing (ABS) protocols for intellectual property in most African countries including Uganda. Even in countries with ABS protocols, there are challenges to equitable sharing of indigenous knowledge (Van Overwalle, 2005; Chikombero and Luseba, 2010). ABS’s objective is to make biodiversity a private good with market opportunities. However, ABS legislation is expensive, unnecessarily bureaucratic, and usually at risk of biopiracy (Richerzhagen, 2007). Traditional knowledge doesn’t fit in the jurisdictions of the patent protection laws (Richerzhagen, 2007). Patent restrictions only apply to processed materials and not to raw materials (Van Overwalle, 2005). Even with set biodiversity authorities, the concept and scope of biodiversity is not clear cut since it is a multi-agency structure (Siebenhüner et al., 2005). The Convention on Biological Diversity (CBD) is non-specific (Siebenhüner and Suplie, 2005). The International Treaty on Plant Genetic resources for food and agriculture does not cover all crops complicating ABS design for some plants (Richerzhagen and Virchow, 2007). There is a need for a government agency to be responsible for granting access permits to companies and international organizations because the local communities are not able to trade this resource (Chikombero and Luseba, 2010). Communities ought to benefit from their ideas but the challenge is the long time lag to confirm research (Richerzhagen and Holm-Mueller, 2005).

## 4 Discussion

### 4.1 Emerging Poultry Anthelmintic Resistance and Plant-Anthelmintics

**FIGURE 8:**
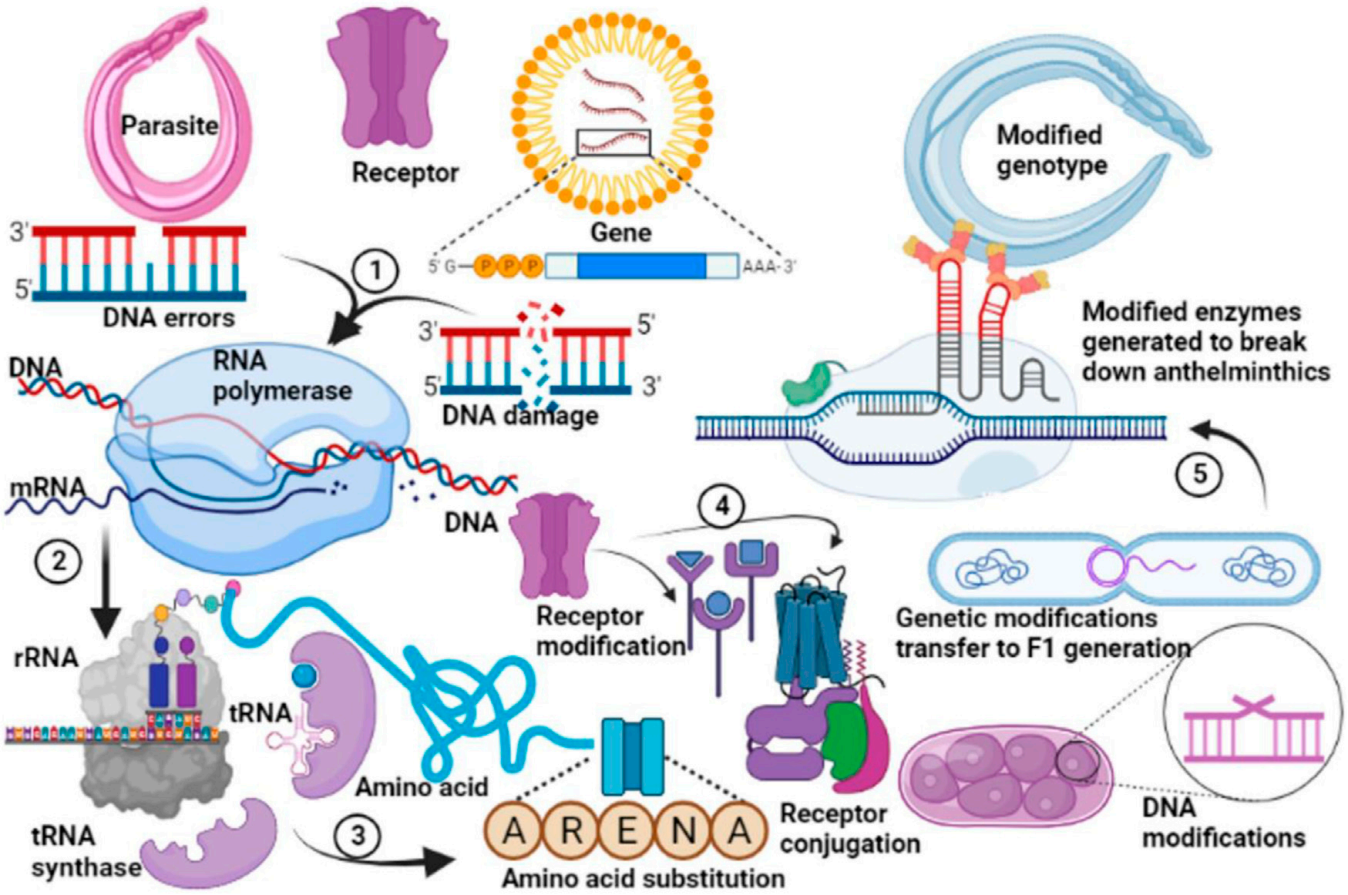
General mechanism of resistance to anthelmintic drugs. Genetic modifications in the parasite occur following decades of application of allopathic anthelmintics. DNA replication errors subsequently promote evolutionary changes in the gene of the parasites to form stable DNA (1). This undergoes transcription (2), and translation (3) with substitution of primary amino acids associated with susceptibility with those which favour resistances against anthelmintics. Subsequent protein modifications (4) favour expression of receptors which inhibit or reduce anthelmintic binding thus protecting the parasite from anthelmintic action. These genetic modifications are transferred to the offspring, favouring selective evolutionary changes which produce metabolic enzymes which degrade anthelmintics (5). A modified genotype is subsequently created which produces subsequent offsprings which are completely resistant to anthelmintics.

Anthelmintic resistance emerges when worms lose sensitivity to a chemical that is known to be lethal to them at the recommended concentration (Beech et al., 2011; Von Samson-Himmelstjerna and Blackhall et al., 2005). There are multiple reports of emerging anthelmintic resistance (Giri and Roy, 2015; Tarbiat, 2018) but studies in low and middle-income countries are scarce. Anthelmintic resistance is a common phenomenon (Kaplan and Vidyashankar, 2012; Whittaker et al., 2017) as a result of evolutionary modifications which take place following chronic exposure to an agent (Figure 8). Because of frequent and/or unlimited use of anthelmintics, resistance is increasing in several countries (Lalthanpuii and Lalchhandama, 2020), although this continues to be under-reported in resource-limited countries probably due to a lack of clear policies for the promotion of biomedical research. Nicotinic agonists such as levamisole become ineffective due to altered drug targets but the resistance mechanism is not fully understood (Köhler, 2001). Levamisole resistance may be linked to change in the nicotinic cholinergic receptor channels among resistant nematodes (Prichard, 1994). Changes in the nicotinic Acetylcholine Receptor (nAchR) subunit encoding genes are associated with Levamisole resistance (Köhler, 2001). Studies of various nAchRs by patch-clamp technique showed variations in Levamisole activated receptor channel currents for levamisole sensitive and levamisole resistant isolates of nematodes (Robertson et al., 1999; Martin et al., 2012). Resistant groups showed a lower number of activated receptor channels (Robertson et al., 1999). Levamisole receptors were observed to be deactivated in resistant isolates (Qian et al., 2008) resulting in a shift in the proportion of nAchR subtypes towards the less sensitive to levamisole (Köhler, 2001).

Macrocyclic lactones such as ivermectin are agonists of inhibitory chloride channels activated by glutamic acid (Geary and Moreno, 2012; Wolstenholme and Kaplan, 2012). Resistance to Macrocyclic lactones is not fully understood although various studies have inferred that it is likely genetically controlled (Gill and Lacey, 1998; Jambre et al., 2000). Resistance develops when mutations take place concurrently in not less than three genes that encode GluCl alpha-type subunits (Wang et al., 2010). It is possible mutations in genes other than those that encode GluCl that lead to resistance (Holden-Dye and Walker, 2006). The mutations lead to physiological changes in the drug target which is the pharyngeal muscle (Keane and Avery, 2003). Ivermectin resistance is also linked to the alteration of the ivermectin receptors (Prichard, 1994).

Benzimidazole resistance is associated with a change in β-tubulin genes (Roos, 1997); precisely substitution of specific amino acids in β -tubulin (Beech et al., 2011). Unregulated use of anthelmintics increases selection for resistance (Van Wyk, 2001; Coles, 2005). Resistant isolates emerge when the administration of benzimidazoles is done when the number of worm eggs and larval stages in the environment is low (Köhler, 2001). This leads to a change in gene expression and subsequent cross anthelmintic resistance (Von Samson-Himmelstjerna and Blackhall, 2005). *Ascaridia galli* is completely susceptible to benzimidazoles (Tarbiat, 2018), demonstrating selective species resistance in the helminths. However many helminths have genetic advantages that leverage their anthelmintic resistance (Shalaby, 2013). Changes in the β -tubulin genes leads to receptor loss or decreased affinity of the binding site for benzimidazole (Lubega and Prichard, 1990).

The poultry industry suffers direct and indirect losses due to anthelmintic resistant varieties of worms (Raza et al., 2016). It is reported that the effects of the increasing global warming are worsening the development of resistance to various anthelmintics (Yazwinski et al., 2013). Medicinal plants are becoming realistic alternatives against helminths that have proven resistant to synthetic anthelmintics (Mahdi et al., 2019). For example, yeast particle encapsulated terpenoids are effective against albendazole-resistant helminths (Mirza et al., 2020). However, the reasons why certain ethnomedicines may be effective against helminths resistant to synthetic anthelmintics are not fully known. The mechanism of action of plant phytochemicals may sometimes be similar to that of synthetic anthelmintics. The action of some saponins is said to be similar to praziquantel (Wang et al., 2010). The plant phytochemicals and their metabolites are diverse with many that need to be tested against the resistant helminths.

### 4.2 Preparation Methods of Ethnoveterinary Anthelmintics

Plant parts commonly used include leaves, barks, and roots (Yirga, 2012; Ritter et al., 2012). Freshly prepared plant parts are more frequently used than dried preparations (Eshetu et al., 2015). Preparation methods are either the traditional on farm practices or the industrial laboratory for plant-based medicines. Traditional on farm methods of preparation can be infusion, decoction and ground fresh materials (McGaw et al., 2007) and macerations (Table 2). Decoctions of barks of *Anogeissus leiocarpus* and *Khaya senegalensis* had anthelmintic potential (Hammond et al., 1997). Traditional maceration methods involve dipping the plant parts in a solvent; the harvested ethno-compound depends on the nature of the solvent and the solid to a solvent ratio (Ćujić et al., 2016).

Industrial laboratory methods can be simulated macerations (Khafagi and Dewedar, 2000; González-Manzano et al., 2004), by use of maceration enzymes (Bautista-Ortín et al., 2005). Extraction time, temperature, and concentration of extraction solvent determine the content of the extract (Chew et al., 2011). Multiple solvents can be used sequentially from the least polar to the most polar. When the polarity of the solvent is almost equal to that of the solute there is a better yield of the extract because the solute dissolves well (Altemimi et al., 2017).

Ethnoveterinary compounds can also be harvested by microwave-assisted extraction (Rafiee et al., 2011). This involves the use of a magnetic field and an electric field to cause extraction. The method is fast and uses less volume of the solvent (Altemimi et al., 2017). Ultrasonic-assisted extraction methods use a particular wavelength that distorts the plant cell wall and enables the solvent to penetrate plant cells (Lianfu and Zelong, 2008; Tian et al., 2013). Smashed samples which are well mixed with a solvent are monitored in an ultrasonic bath.

Phenolics are best harvested by mixing air-dried plant parts with the solvents (Djeridane et al., 2006). Alkaloids are harvested using gold seal-based pressurized hot water, reflux, or ultrasonic methods (Mokgadi et al., 2013). Saponins are harvested by accelerated solvent extraction, ultrasonic-assisted extractions, or pressurized low polarity water methods (Guclu-Ustundag and Mazza, 2007). To harvest terpenoids, the supercritical fluid extraction method was better than the soxhlet extraction methods when n-hexane or ethyl alcohol solvent were used (Kristo et al., 2001). Papaya metabolomes are harvested using the soxhlet extraction methods (Bah et al., 2006; Nakamura et al., 2019). Isoflavones could be harvested by soxhlet, shaking, vortexing, sonication, pressurized liquid extraction, or stirring all with a solvent (Luthria et al., 2007). Artemisinins are harvested by microwave-assisted extraction with the diameter of materials at most 0.125 mm (Hao et al., 2002). Modern preparations of the plant products ought to simulate the traditional preparation which initiated the claim, however, this is not always practical. The solvents and purification equipment are very expensive for low resource country early-career scientists interested in ethnoveterinary anthelmintics.

### 4.3 Traditional Use of Plants in Controlling Poultry Helminths

Use of plant-based ethnomedicines in managing poultry helminth infections has been well documented for other parts of the world but is scanty for sub-Saharan Africa. However their use is widespread as alternative to conventional medicines or when it’s the only accessible option for attempting treatment (Nyamanga, Suda, and Aagaard-Hansen, 2008).

Decoctions of *Cannabis sativa L* leaves (Iqbal et al., 2006; Hartady et al., 2021), *Sanseveria nilotica* Baker leaves (Nabukenya et al., 2014), *Carica papaya L* roots, *cassia occidentalis L* either roots or leaves and *Boerhivia diffusa L* leaves are used against poultry helminths. These decoctions are prepared by boiling plant parts in water and left to simmer, cooled and orally administered to poultry. Roots or barks of *Aloe vera L* are used in making decoctions for use against poultry helminths (Iqbal et al., 2006; Hartady et al., 2021).

Infusions of *Cannabis sativa L* leaves and *Carica papaya L* roots are also used against poultry helminths (Nabukenya et al., 2014), infusions of *Nicotiana tabacum L* leaves are also used for the same purpose (Iqbal et al., 2006; Hartady et al., 2021). The infusions are made by soaking plant parts in water which may be hot or cold. The plant parts are left to stand in the water to allow the plant active compounds to move into the water. The use of plant infusions in traditional poultry medicine is highly acceptable by communities although such practices need substantiation (Gueye, 1999).

Concoctions of various plants can be used to generate a blanket treatment against gastro-intestinal disorders (Nalubega, 2010). The use of plant combinations is the most used in poultry plant based treatments because they target many disease causing agents (Haniarti et al., 2019; Moreki, 2012). Various plants are combined together and crushed to generate juice for treatment or crushed and preserved for making infusions in the future.

In Uganda (Africa), traditionally prepared materials are also available as dried plant parts for infusions which are sold in packets as general poultry care products against gastro-intestinal disorders. The details of the composition and method of preparation usually remain secretive as a source of livelihood for the herbalists in the communities. However it is thought that some other non-plant materials are added to the preparations as a custom or procedure of getting the most desired outcomes or preventing hazardous outcomes. Materials like unspecified wood ash, common salt, cooking oils and alcohol are said to be sometimes added to the plant materials. Traditional use of plants in poultry medicine is challenged by lack of clear policies on use and protection of intellectual property. Plant compositions are affected by many factors like season of year and edaphic factors, which affect standardization of use. However industrial research on the traditionally used plants may provide links to developing new anthelmintics.

### 4.4 Future Research

Studies on the pharmacokinetics and dynamics of various ethno-anthelmintics are needed. Profiling of ethno-anthelmintics and susceptibility patterns of thelmintic species would help guide policy for the promotion of these products. Pharmacological experiments would help improve vigilance on the use of ethnomedicines for animal production and help reverse the current trend of drug abuse by ethnomedicinal practitioners (for example in Uganda (Schillhorn and Van Veen, 1997). Phytochemical analyses have been developed (Heinrich, 2008) but need to be applied to all-natural medicines. The metabolomics of several plant candidates need to be undertaken and a systematic ethnoveterinary anthelmintic database created. There is a need for collaborations between biomedical scientists to move study findings from the laboratory to the field. Studies on the anthelmintic synergy of different plant species combinations or ethnomedicine and synthetic combinations need to be considered.

## 5 Conclusion

Ethnoveterinary anthelmintics are an inadequately explored alternative to synthetic anthelmintics. They are an option in the era of organic farming and reducing synthetic drug residues in poultry products. In some cases, they have been claimed to be effective against helminths resistant to synthetic anthelmintics. Though these plant alternatives may be cheap and accessible, they have limitations. The efficacy and toxicity of most of the plant alternatives are usually controversial or completely unknown. Most inferences have been based only on *in vitro* assays and very limited *in vivo* assays have been reported in poultry. Very few species of helminths have been used as models (usually *Ascaridia galli*), it’s not known whether all other helminths would respond like the selected models. Licensing and regulating of ethnoveterinary anthelmintics can be leveraged by robust studies of these plant alternatives in poultry medicine. Ethnoveterinary anthelmintics also offer the basis for the new generation of anthelmintics made as a combination with nanoparticles. The identified bio-active molecules effective against poultry helminths could be studied to guide the development of new generations of anthelmintics. Several opportunities could be achieved from ethnomedicinal alternatives by exploring synergistic possibilities with conventional anthelmintics.

## Abbreviations

HPLC, High Performance Liquid Chromatography; GluCl, Glutamate-gated Chloride; CBD, Convention on Biological Diversity; ABS, Access and Benefit Sharing; IP, Intellectual Property; h, hour; Spp, Species; BITC, Benzyl Isothiocyanate; DNA, Deoxyribonucleic acid; CT, Condensed Tannins; EVM, Ethnoveterinary Medicine; ABZ, Albendazole; ABZSO, Albendazole sulfoxide; ABZSO2, Albendazole sulphone; T1/2, Half life; µg, Micro-grams; Kg, Kilograms; ADI, Acceptable Daily intake; EC, European Commission; BW, Body weight; SDG, Sustainable Development Goal.
